# Quality Improvement in Coronary Care: Analysis of Sustainability and Impact on Adjacent Clinical Measures After a Swedish Controlled, Multicenter Quality Improvement Collaborative

**DOI:** 10.1161/JAHA.112.000737

**Published:** 2012-08-24

**Authors:** Rickard Carlhed, Christina Bellman, Mats Bojestig, Leif Bojö, Anette Peterson, Bertil Lindahl, the Quality Improvement in Coronary Care (QUICC) Study Group

**Affiliations:** 1 Department of Oncology, Central Hospital, Karlstad, Sweden (R.C.); 2 Department of Clinical Physiology, Central Hospital, Karlstad, Sweden (L.B.); 3 Department of Medical Sciences and Uppsala Clinical Research Center, Uppsala University, Sweden (C.B., B.L.); 4 Department of Healthcare, Jönköping County Council, Sweden (M.B., A.P.)

**Keywords:** acute myocardial infarction, guideline adherence, quality improvement, quality indicators, registries

## Abstract

**Background:**

Quality Improvement in Coronary Care, a Swedish multicenter, controlled quality-improvement (QI) collaborative, has shown significant improvements in adherence to national guidelines for acute myocardial infarction, as well as improved clinical outcome. The objectives of this report were to describe the sustainability of the improvements after withdrawal of study support and a consolidation period of 3 months and to report whether improvements were disseminated to treatments and diagnostic procedures other than those primarily targeted.

**Methods and Results:**

Multidisciplinary teams from 19 Swedish hospitals were educated in basic QI methodologies. Another 19 matched hospitals were included as blinded controls. All evaluations were made on the hospital level, and data were obtained from a national quality registry, Swedish Register of Information and Knowledge About Swedish Heart Intensive Care Admissions (RIKS-HIA). Sustainability indicators consisted of use of angiotensin-converting enzyme inhibitors, lipid-lowering therapy, clopidogrel, low-molecular weight heparin, and coronary angiography. Dissemination indicators were use of echocardiography, stress tests, and reperfusion therapy; time delays; and length of stay. At the reevaluation period of 6 months, the improvements at the QI intervention hospitals were sustained in all indicators but 1 (angiotensin-converting enzyme inhibitor). Between the 2 measurements, the control group improved significantly in all but 1 indicator (angiotensin-converting enzyme inhibitor). However, at the second measurement, the absolute adherence rates of the intervention hospitals were still numerically higher in all 5 indicators, and significantly so in 1 (clopidogrel). No significant changes were observed for the dissemination indicators.

**Conclusions:**

The combination of a systematic QI collaborative with a national, interactive quality registry might lead to substantial and sustained improvements in the quality of acute myocardial infarction care. However, to achieve disseminated improvements in adjacent clinical measures, those adjacent measures probably should be made explicit before any QI intervention. **(*J Am Heart Assoc*. 2012;1:e000737 doi: 10.1161/JAHA.112.000737.)**

## Introduction

During the past 2 decades, several international studies have shown ongoing, cumulative improvements in the quality of care, as well as decreased short-term death rate, for patients with acute myocardial infarction (AMI).^1–3^ However, the same studies also show that there still is considerable potential for further improvements and that the implementation rate of new evidence-based diagnostic procedures and treatments is slow.^1–3^

The Quality Improvement in Coronary Care (QUICC) study^4^ was initiated in 2002 as an attempt to improve the care of patients with AMI in Sweden. This was a national controlled study in which 19 hospitals of varying sizes participated in a quality-improvement (QI) collaborative based on the “Breakthrough” model of improvement, and 19 matched hospitals served as controls.^5–6^ Five established, guideline-derived quality indicators were used to assess the quality of AMI care: use of lipid-lowering therapy, angiotensin-converting enzyme inhibitors, and clopidogrel at discharge; and use of heparin or low-molecular-weight heparin and coronary angiography during hospitalization. All 5 quality indicators showed significant improvements relative to baseline in the 19 hospitals participating in the QI program. Compared to the 19 control hospitals, the improvements were significantly higher for all indicators but 1.^4^ In a subsequent article, it was demonstrated that the increased guideline adherence rates were associated with a decrease in cardiac morbidity and mortality rates.^7^

Nevertheless, success in a relatively short timeframe is one thing, but a time- and resource-consuming QI program like the one in the QUICC study also should lead to sustained improvements, as stated by Øvretveit^8^: “Results are important, but do not guarantee continuation.” Furthermore, it is unknown whether the used QI model influences, positively or negatively, the use of other important but not primarily targeted diagnostic procedures and treatments or critical time delays.

Therefore, in accordance with the QUICC study protocol, we prospectively evaluated the sustainability of the initial improvements in the 5 targeted quality indicators and whether positive effects on other, not primarily targeted, key measurements could be demonstrated.

## Methods

### Setting and Patient Selection Criteria

All patients admitted to the coronary care unit with a discharge diagnosis of AMI (I21) according to the *International Classification of Diseases, 10th revision* and an age <80 years were included in the study. The age limit was introduced to reduce confounding by comorbidities not recorded in the registry and to avoid selection bias due to between-hospital variations in the tendency to admit patients of the highest age to the coronary care unit.

All Swedish hospitals with coronary care units participating in the Swedish quality registry for acute coronary care, RIKS-HIA (73 of 78 hospitals in total in 2003), were invited to participate in the study. Of those, 21 hospitals accepted the invitation to participate. However, because 1 hospital never joined the collaborative and 1 hospital was closed early during the study period, the evaluations are based on data from 19 hospitals. The intervention hospitals were subdivided into 4 strata on the basis of presence or absence of in-house coronary angiography, and a historical, composite treatment performance level above or below a national average. Another matching 19 Swedish hospitals were selected manually and subdivided into the same 4 strata and were used as blinded controls.

### Design of the Intervention

The design of the QUICC study and the results from the initial evaluation have been described in detail previously.^4–5^ In short, multidisciplinary teams from 19 volunteering Swedish hospitals met at 2 or 4 training sessions during which they were educated in basic QI methodologies. The teams also were trained in how to optimize their use of the modified RIKS-HIA registry. The teams were guided in how to generate real-time performance feedback and how to use it to improve their care processes. The training sessions were held during a 6-month period, and between these gatherings, each team developed and implemented strategies for improving AMI care at their hospital. At the subsequent sessions, the different teams presented their ideas for improvement, their achievements, and their experiences of problems and obstacles.

After the initial 6-month learning and implementation period, the performances of the intervention and control hospitals were investigated during the ensuing 12 months (measurement period 1 [M1]: May 1, 2003, to April 30, 2004).

The quality of AMI care was evaluated according to 5 quality indicators: predischarge prescriptions of lipid-lowering therapy, angiotensin-converting enzyme inhibitors, and clopidogrel; use of heparin or low-molecular-weight heparin during hospitalization; and finally, coronary angiography performed before discharge. These performance measures were all evidence based and well established and had been recommended in international AMI guidelines.^9–10^ For each of these indicators, the evaluations were based on ideal patients—that is, patients who had indications for but lacked contraindications against each specific treatment.^4^ Other well-established quality indicators, such as use of β-blockers and aspirin, were not evaluated because of the fact that the prestudy treatment levels were known to be excellent and hence not possible to further improve.

To analyze if the assumed improvements were sustained over time, a new evaluation of the performance levels was made after a consolidation period of 3 months, before which all support from the study management group was withdrawn. The reevaluation period (M2) extended over 6 months, from August 1, 2004, to January 31, 2005.

The second purpose of this follow-up study was to evaluate if any effect, positive or negative, could be demonstrated for other, adjacent clinical measures not primarily targeted in the QI intervention. The following prespecified clinical measures were evaluated during the baseline measurement period (July 1, 2001, to June 30, 2002) and M1 (May 1, 2003, to April 30, 2004): use of stress test, use of echocardiography, performance of reperfusion therapy (ST-elevation myocardial infarction), time delay from the emergency department to start of reperfusion therapy (ST-elevation myocardial infarction), and length of stay for the initial care episode.

The study was approved by the ethics committee at Uppsala University.

### Data Source and Accuracy

All QUICC centers and control centers continuously entered information into the RIKS-HIA registry about all patients admitted to the coronary care units. For each patient and care episode, about 110 separate parameters were entered into the registry. These parameters comprised demographics, risk factors, previous diseases, examinations, medication, interventions, time delays, and diagnoses. RIKS-HIA has been described more thoroughly elsewhere.^11^

The quality of the data entered into RIKS-HIA is monitored routinely. In 2003, 574 and 572 randomly selected local patient records were verified in the intervention and control hospitals, respectively. The accuracy averaged 89.9% and did not differ between the 2 groups.

### Statistical Analysis

Patients eligible for each measure were “target patients”—that is, patients with indications for and without contraindications to each treatment or intervention under evaluation. Results were collected on a hospital basis, and all further comparisons were made with hospitals as units of analysis.

Within-group differences from one measurement period to another were evaluated with 2-tailed Student *t* tests for paired data. For comparisons between the control and intervention groups, 2-way ANOVA tests were used to account for the stratification. In all tests, *P* values <0.05 were considered statistically significant. Statistical analyses were performed with statistical software (SPSS Statistics, version 20.0.0, IBM Corporation, Somers, NY).

## Results

### Hospital and Patient Characteristics

During the first measurement period, M1, 3848 and 3037 AMI patients <80 years of age were cared for at the 19 intervention hospitals and the 19 control hospitals, respectively. For the second measurement period, M2, the corresponding numbers of AMI patients were 1929 and 1472, respectively.

There were no significant differences in baseline patient characteristics between the 2 hospital groups, except that the intervention hospitals had a slightly smaller proportion of patients with previous myocardial infarctions during both M1 and M2 and also a lower prevalence of diabetes mellitus during M2 (Table 1).

**Table 1.** Baseline Characteristics During the Measurement Periods

	M1 (12 Months)		M2 (6 Months)	
	Control	Intervention	Control	Intervention
AMI patients, mean number/hospital	159.8	202.5	77.5	101.5
Age, mean, y	66.6	66.1	65.7	65.7
Women, %	30.5	31.4	32.5	31.2
Previous AMI, %	30	26.3	28.3	25.3
Diabetes mellitus, %	21.3	21.2	23.8	20.1
Hypertension, %	40.8	39.2	43.6	43.7
Treated hyperlipidemia, %	27.7	26.6	28.9	30.3
Smoking (previous or current), %	55.1	57.1	56.3	58.5

AMI indicates acute myocardial infarction.

### Sustainability of Improvements

**Figure 1.**
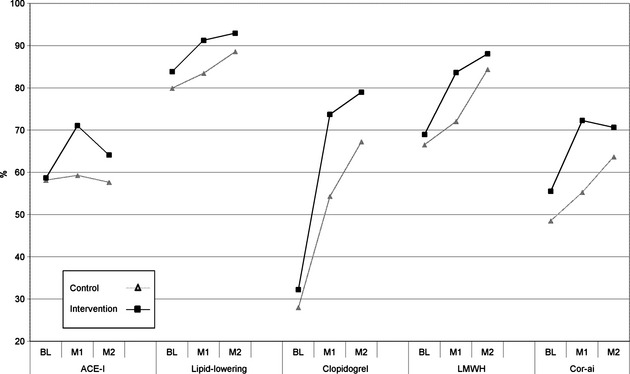
Mean adherence rates at baseline (BL), M1, and M2. ACE-I indicates angiotensin-converting enzyme inhibitor; LMWH, low-molecular-weight heparin; and Cor-ai, coronary angiography.

The Figure 1 gives a graphical presentation of the mean adherence rates for the intervention and control hospitals at baseline, M1, and M2, respectively**.** In Table 2, the corresponding mean adherence rates in M1 and M2, as well as the statistical significance for the intragroup changes from M1 to M2, are presented numerically. From M1 to M2, the adherence rates for the intervention hospitals were sustained or improved for all but 1 indicator (angiotensin-converting enzyme inhibitor). During the same time, an evident catch-up effect was seen in the control group. As an effect, the large mean differences between the hospital groups in M1 had decreased substantially to M2. Still, for all indicators, the absolute adherence rates in M2 were numerically higher in the intervention group, although the difference was only significant for clopidogrel (79.0 versus 67.2%, *P*=0.03) (Table 3).

**Table 2.** Differences in Changes of Mean Adherence Rates From M1 to M2

	Control Hospitals (n=19)				Intervention Hospitals (n=19)			
	M1	M2	Difference, %	*P*	M1	M2	Difference, %	*P*
ACE inhibitors	59.29	57.66	−1.63	NS	71.06	64.11	−6.95	0.01
Lipid-lowering therapy	83.46	88.61	5.15	0.02	91.28	92.96	1.68	NS
Clopidogrel	54.30	67.24	12.94	<0.01	73.73	78.99	5.26	0.09
Heparin/LMWH	72.07	84.38	12.31	<0.01	83.66	88.08	4.42	0.07
Coronary angiography	55.27	63.69	8.42	0.01	72.29	70.66	−1.63	NS

ACE indicates angiotensin-converting enzyme; LMWH, low-molecular-weight heparin.

**Table 3.** Differences Between Hospital Groups in Absolute Adherence Rates: M2

	Control Hospitals (n=19), %	Intervention Hospitals (n=19), %	*P*
ACE inhibitor	57.66	64.11	0.111
Lipid-lowering therapy	88.61	92.96	0.102
Clopidogrel	67.24	78.99	0.034
Heparin/LMWH	84.38	88.08	0.097
Coronary angiography	63.69	70.66	0.077

ACE indicates angiotensin-converting enzyme; LMWH, low-molecular-weight heparin.

### Effects on Adjacent Clinical Measures

From baseline to M1, no significant between-group differences could be demonstrated in the changes of the 5 preselected clinical measures not primarily targeted in the QI program (Table 4). The observed decrease in the use of stress tests in both groups is explained by concurrent guideline modifications that recommended routine coronary angiography without a preceding stress test in non–ST-elevation AMI.

**Table 4.** Effects on Adjacent Clinical Measures: Baseline to M1

Differences, Baseline to M1	Baseline		M1		Differences		
	Control	Intervention	Control	Intervention	Control	Intervention	*P*
Stress test, %	30.3	24.4	17.2	12.4	−13.1	−12.0	NS
Echocardiography, %	59.5	64.8	59.3	68.6	−0.2	3.8	NS
Reperfusion therapy, %	71.3	71.8	68.8	71.3	−2.5	−0.5	NS
Mean delay (ED to thrombolysis), min	53.8	65.0	64.8	57.1	11.0	−7.8	0.08
Mean length of stay, d	6.85	6.41	6.89	6.31	0.04	−0.1	NS

ED indicates emergency department.

## Discussion

In previous publications, we have shown that the QUICC intervention was successful in reaching its primary aims, with documented improvements in adherence to the national AMI guidelines as well as improvements in clinical outcome.^4,7^ In the present report, we show that the improvements were sustained over time. On the other hand, no improvements were found in other clinical measures besides the explicit ones.

In this present study, we addressed important questions formulated by some leading international researchers with experience from the collaborative model of QI: “If any improvements made are not maintained or spread after the collaborative, it is questionable whether a collaborative is worth the cost.”^12^ Undoubtedly, to justify a later expansion of a time- and resource-demanding QI effort such as the QUICC intervention, it has to be shown that the results are maintained. Up to now, evaluations of the sustainability of the effects of QI interventions in the field of acute cardiovascular disease have been sparse.

Our findings that the initial improvements in guideline adherence were sustained over time are in accordance with the experiences gained from the follow-up study of the Get With The Guidelines–Coronary Artery Disease program,^13^ in which it was shown that initial improvements in guideline adherence for 6 measures (aspirin at arrival and discharge; β-blocker at arrival and discharge; angiotensin-converting enzyme inhibitor for left ventricular systolic dysfunction; and smoking cessation counseling) were sustained over 3 consecutive annual measurements. Moreover, our finding of a catch-up effect in the control group is also in accordance with the narrowing between the study groups that was seen in the Get With The Guidelines–Coronary Artery Disease program during their follow-up period, although the Get With The Guidelines–Coronary Artery Disease intervention hospitals maintained higher adherence rates than those of the nonintervention hospitals for the entire follow-up period.^13^

A possible explanation for the pronounced improvements in the control group between the 2 measurement periods in the present study might be that RIKS-HIA registry data reflecting performance levels for individual hospitals were made public for the very first time during the interval between the 2 measurements. The succeeding debate in public media about national inequalities in the quality of AMI care likely increased the interest of most healthcare providers in their own quality of care. Another possible explanation is that the QI activities during the QUICC project in the 19 intervention hospitals (involving one fourth of all Swedish hospitals managing AMI patients) consequently also increased interest in the quality of care of AMI patients in the remaining Swedish hospitals. In either of these circumstances, a spillover effect is probable, an assumption that is in line with the experience of others who have shown that national quality campaigns with open-access resources can have a substantial spillover effect on nonenrolled hospitals.^14^

The second important question dealt with in this study was whether the quality activities targeting the 5 explicit quality indicators also would catalyze improvements in other guideline-derived measures. Here, it was somewhat disappointing that no positive effect was seen on any of the 5 secondary clinical measures under scrutiny. This indicates that the quality activities in the local organizations had been confined to the 5 explicit quality indicators during the QI work. Unfortunately, we have no information about whether the interventions hospitals started new QI work after completion of the QI work mandated by the study. Our findings are somewhat in contrast to a 1-year follow-up survey of a collaborative in a different medical area (fall injury prevention) but with the same foundation as ours: the Breakthrough Model of Improvement. There, Neily et al^15^ found that 85% (29 of 34) of their teams reported that they had begun QI work on new topics; however, the study did not indicate whether any actual improvement was achieved.

Our study has some important limitations. First, the teams of the included hospitals all volunteered to participate, which could have led to self-selection bias. This in turn might have promoted superior improvements in the intervention group because of a greater eagerness to improve. Despite this, there were no baseline differences in treatment adherence rates between the 2 groups, implying that any potential difference in “eagerness to improve” does not by itself translate into superior quality of care. This eagerness thus seems to have to be accompanied by some type of systematic QI activities.

Second, our registry-derived data might be regarded as less reliable than data gained from a randomized, controlled study. Nevertheless, by means of thorough and continuous monitoring activities of the RIKS-HIA registry, we are confident that the data are both reliable and representative. Third, the time span of our follow-up was rather narrow; it would have been advantageous to have a longer follow-up period. Unfortunately, this was not feasible because other national AMI QI campaigns were launched shortly after our intervention, which would have made it very difficult to differentiate the effects of the separate interventions. Fourth, because of the fact that at baseline, many of the low-volume hospitals had not yet implemented the recommended routine to treat patients with ST-elevation AMI with primary percutaneous coronary intervention, it unfortunately was not possible to analyze the prespecified clinical measure “time delay from the emergency department to primary percutaneous coronary intervention” in a meaningful way. Finally, in this report, we have only shown that the improvements were lasting, and we have not answered why they were sustained—that is, we have not determined what factors were associated with the observed sustainability. An attempt to answer this would certainly be an interesting aim for future studies of QI initiatives.

## Conclusions

To conclude, we have found that the initial improvements in AMI guideline adherence were sustained over time and in some aspects even were further augmented. However, the local QI activities that brought about these improvements did not generate any advances in other adjacent clinical measures.

An important lesson from this evaluation is that before a QI project is started, a meticulous selection of and restriction in number of targeted measures should be done. Identification of the most important areas in need of improvement is crucial, because if areas are not included, improvements will likely not occur in the omitted areas. On the other hand, attempting to cover too many measures is probably counterproductive because the barriers to implementation of each guideline recommendation might differ and therefore require different remedies.^16^
